# Enrolment patterns in a randomized controlled trial of probiotics in critically ill patients: a retrospective analysis of the PROSPECT trial

**DOI:** 10.1186/s13063-024-08701-w

**Published:** 2024-12-27

**Authors:** Alyson Takaoka, Jennie Johnstone, François Lauzier, Diane Heels-Ansdell, Megan Davis, Nicole Zytaruk, Erick Duan, Joanna Dionne, Lois Saunders, Yaseen M. Arabi, John Marshall, Lehana Thabane, France Clarke, Lori Hand, Marie-Helene Masse, Bram Rochwerg, Lauralyn McIntyre, Martin Girard, Andreas Freitag, Tim Karachi, Deborah J. Cook, For The PROSPECT Investigators, Mary Copland, Shelley Anderson-White, Melissa Shears, Kristine Wachmann, The Canadian Critical Care Trials Group, Lisa Patterson, Marat Slessarev

**Affiliations:** 1 Queen’s University, Kingston, Canada; 2 University of Toronto, Toronto, Canada; 3 Université Laval, Québec City, Canada; 4 McMaster University, Hamilton, Canada; 5 McMaster University, Hamilton, ON Canada; 6 King Saud bin Abdulaziz University Health Sciences and King Abdullah International Medical Research Center, Riyadh, Saudi Arabia; 7 Université de Sherbrooke, Sherbrooke, Canada; 8 University of Ottawa, Ottawa, Canada; 9 Université de Montréal, Montréal, Canada

**Keywords:** Randomized trial, Critical illness, Enrolment, Screening

## Abstract

**Background:**

Understanding site-related factors that influence enrolment within multicenter randomized controlled trials (RCT) may help reduce trial delays and cost over-runs and prevent early trial discontinuation. In this analysis of PROSPECT (*Pro*biotics: Prevention of *S*evere *P*neumonia and *E*ndotracheal *C*olonization *T*rial), we describe patient enrolment patterns and examine factors influencing site-based monthly enrolment.

**Design:**

Retrospective analysis of a multicenter RCT.

**Methods:**

The PROSPECT multicenter RCT enrolled patients in the main trial from July 2015 to March 2019. We documented site characteristics and trial metrics including data from the methods center tracking documents, site-level data at trial initiation, screening logs submitted by research coordinators, and prospectively collected case report forms. In this retrospective analysis of trial data, we analyzed enrolment patterns across sites using negative binomial regression to explore the association between monthly enrolment rate accounting for number of ICU beds, site characteristics, and trial metrics.

**Results:**

Overall, 41 sites enrolling 2365 patients in the PROSPECT main trial were analyzed. After accounting for number of beds in each ICU, site launch early in the trial was associated with higher monthly enrolment rates, but time to first enrolment and research coordinator experience was not. We observed considerable variability in the number of active screening months and enrolment rates across sites.

**Conclusion:**

These findings highlight the complexity of recruitment dynamics in critical care RCTs and emphasize the need for tailored approaches to trial planning and execution.

**Trial registration:**

PROSPECT (Probiotics: Prevention of Severe Pneumonia and Endotracheal Colonization Trial): NCT02462590 (registered June 2, 2015).

## Background

Projected patient enrolment rates in multicenter randomized clinical trials (RCTs) are often over-estimated, leading to delayed trial completion, cost over-runs, and applications for grant extensions. Furthermore, low patient recruitment is the one of the most frequent reasons for trial discontinuation [1, 2].

While site selection for multicenter trials often hinges on pragmatic considerations, including interest in participating, local case-mix alignment with eligibility criteria, and past collaborations with the coordinating team [3], identifying sites that may face enrollment challenges before commencement may allow trial methods centers to extend early support. This could include sharing recruitment strategies and providing individualized mentorship to enhance site capacity, ensuring success in the current trial and fostering future collaboration [4].

Site-level factors influencing patient enrollment are unclear, and published trials seldom report on enrolment patterns or their determinants. Our study addresses this gap using a retrospective analysis of a randomized trial testing the effects of probiotics on infectious outcomes during critical illness (PROSPECT: *Pro*biotics to prevent *S*evere *P*neumonia and *E*ndotracheal *C*olonization *T*rial; NCT02462590) [5] aiming to (1) describe patient enrolment across sites over the course of the trial and (2) examine early site factors which may be associated with monthly enrolment.

## Methods

### PROSPECT trial summary

**Fig. 1:**
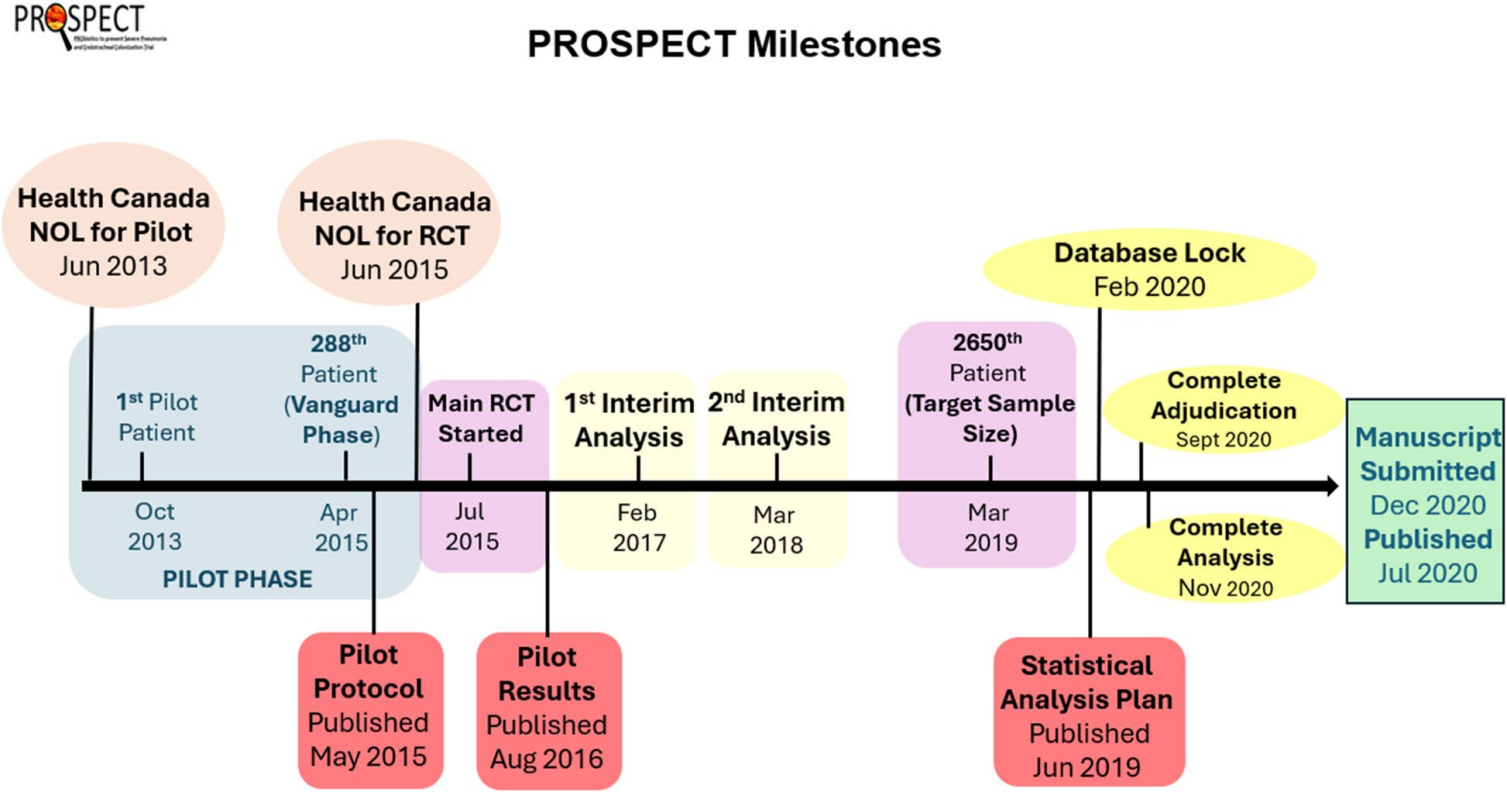
PROSPECT Timeline and Milestones. Timeline of the PROSPECT trial, including major milestones, beginning with pilot trial registration in June 2013 and completing with final manuscript published in July 2020. NOL = no objection letter; RCT = randomized controlled trial

The PROSPECT trial was conducted between July 2015 and March 2019. We enrolled a total of 2653 patients across 44 sites (288 patients in the pilot and vanguard phases, [6, 7] and 2365 patients in the main phase of the trial, which is the focus of this report). There were 42 centers in Canada, 1 in the United States, and 1 in Saudi Arabia. In PROSPECT, we randomized invasively mechanically ventilated patients to receive the study product (1 × 10^10^ colony-forming units of *Lactobacillus rhamnosus* GG) or an identical enteral placebo for up to 60 days, until discharge from ICU [8]. We found that *L. rhamnosus GG* did not reduce the risk of ventilator-associated pneumonia, *Clostridioides difficile* or other infections, or any other clinically important endpoints [5]. We summarize the trial timeline in Fig. 1.

### Study design

In this retrospective analysis of the PROSPECT multicenter RCT, we included data sources from prospectively collected case report forms (CRF) of site-level data captured at site initiation, the methods center’s internal tracking documents, and self-reported screening logs submitted by site research coordinators to the methods center. For each participating site, we extracted the following information: level of care (tertiary care vs. non-tertiary care), number of ICU beds at trial initiation, prior trial engagement (new randomized trial collaboration with coordinating group vs. previous collaboration), participation in the PROSPECT pilot or vanguard trial (yes/no), date of study initiation (Health Canada approval for each Canadian site, and opening in randomize.net for the Saudi Arabian site), date of first patient randomization, and years of ICU research experience of the site’s lead research coordinator (≥ 10 years vs. < 10 years) at trial initiation. Additional metrics included the number of months in which the site was open, total screening months, and total patient enrolment (per month and overall).

### Participating sites and patients

All investigators caring for critically ill adults affiliated with the Canadian Critical Care Trials Group (CCCTG) have the opportunity to participate in trials associated with this consortium, including PROSPECT. Certain sites which previously collaborated with the methods center participated in this trial, as well as additional new sites. Sites engaged serially rather than concurrently, such that the trial was launched with three open sites. On-boarding of additional sites occurred over the course of the trial up a final total of 44 sites. During the trial, three independent centers merged into one hospital institution. For the purpose of this analysis, we focused on early site-level factors possibly predicting enrolment and include the initial three centers but excluded data from the merged center. We excluded two sites that participated in the pilot study but not in the main trial. Therefore, this analysis includes 41 sites and each site was audited or monitored once during the trial [9].

We defined open sites as those with research ethics approval and fully executed contracts. Recruiting sites were defined as those actively screening for patients to enroll which followed an official site initiation visit. A screening month was defined as a month in which at least one patient was enrolled or in which a research coordinator screened ICU patients for trial eligibility during at least 2 weeks.

### Measurements

The primary outcome measure for this study was average monthly enrolment rate per site over the duration of the trial, accounting for the number of ICU beds. This approach avoids the confounding effect of number of ICU admissions and serial site engagement during the trial. As all else being equal, a larger ICU with a greater number of beds and a site with more months to recruit patients could have more enrolment opportunities. We excluded patients enrolled in either the pilot or vanguard phase and included only enrolments from the main trial.

To explore the association between monthly enrolment rate accounting for number of ICU beds, site characteristics, and trial metrics, we were limited to three independent variables to avoid model overfitting [10]. These variables were selected a priori based on the following criteria: (1) hypothesized influence on trial enrolment patterns based on factors identified in the literature, (2) collected prior to the site’s first randomization, and (3) availability in the PROSPECT trial database or internal documents.

We used time (in days) from the site’s regulatory approval to the first randomization to represent the time it takes to begin enrolling patients. In this trial, for Canadian sites, Health Canada’s acknowledgement of receipt of the clinical trial site information was the final regulatory step before sites could enroll patients. For one site in Saudi Arabia, regulatory approval was not required as probiotics in this jurisdiction are not considered a drug. Thus, we used the official opening date in the randomization software, which, comparably, was the last step before recruitment could be initiated after ethics approval.

We divided total RCT time into quartiles and determined within which quartile each site enrolled their first patient (1st quartile of the trial vs. 2nd, 3rd, or 4th quartile of the overall trial recruitment).

Lead research coordinator years of ICU research experience (≥ 10 vs. < 10 years) at the time of Health Canada approval is a binary variable which we used to explore whether more experienced research coordinators supporting trial initiation influenced the average monthly recruitment rate.

### Statistical analysis

We report descriptive statistics of trial and site characteristics. For continuous variables, we report means and standard deviations (SD) if normally distributed and medians and interquartile ranges (IQR) if not.

We performed multivariable negative binomial regression to estimate the association between predictor variables (time to first randomization, quartile of site engagement, research coordinator years of experience) and the primary outcome (site enrolment rate). We report negative binomial regression results as incident rate ratios (IRR) and 95% confidence intervals (CI), with associated *p*-values. All *p*-values are reported to three decimal places. We set the criterion for statistical significance at alpha = 0.05. All analyses were performed using the Statistical Analysis System version 9.4 (SAS Institute Inc., Cary, North Carolina, USA).

### Ethics

The PROSPECT RCT was approved by each hospital’s research ethics board using an a priori consent model wherein written informed consent was obtained from each patient or their substitute decision maker prior to enrolment. PROSPECT was conducted in accordance with the principles of Good Clinical Practice following the Tri-Council Guidelines [11]. We did not require ethical approval to carry out this post-hoc analysis since data included only site-level variables with no patient-level data or identifiers.

## Results

### Trial characteristics

We summarize trial characteristics in Table 1.

**Table 1:** Trial characteristics

**Trial characteristics**	
Recruitment period	
Pilot/vanguard trial	October 2013–August 2014
Main trial	July 2015–March 2019
Number of participating ICUs	
Pilot/vanguard trial	15^a^
Main trial	44^b^
Number of patients	
Pilot/vanguard trial	288
Main trial	2365
Total	2653

In this table, we present trial characteristics including recruitment period, number of participating ICUs, and number of patients enrolled

^a^13 of 15 pilot centers continued to participate in the main trial

^b^The current analysis includes 41/44 centers. We excluded data from 1 merged site and 2 sites in the pilot study that did not participate in the main trial

### Site characteristics

We included 41 sites that participated in the main PROSPECT RCT in our analysis. We summarize combined site variables in Table 2.

**Table 2:** Site characteristics

**Site characteristics**	
Previous collaboration with trial coordinating group, *n* (% yes)	32 (78.0)
Number of ICU beds	
Mean (SD)	25.6 (10.2)
Median (IQR)	24 (7)
Range	10–60
Pilot trial participation, *n* (%)	13 (31.7)
Tertiary care ICUs, *n* (%)	34 (82.9)

In this table, we present characteristics of 41 sites participating in the main trial including previous collaboration with trial coordinating group, number ICU beds, pilot trial participation, and type of ICU

*SD* standard deviation, *IQR* interquartile range

### Aim 1: Trial progress metrics

Trial progress metrics are summarized in Table 3. From the first (July 2015) to the last (March 2019) month of patient enrolment, there were a total of 45 possible screening and enrolling months. Across sites, there were a cumulative 1093 total screening months, for a mean (SD) of 26.2 (10.3) screening months per site. The number of screening months ranged from 9 to 45 months per site. The mean overall enrolment rate across all sites was 52.5 (25.3) patients per month for the whole trial.

**Table 3:** Main trial screening and enrolment metrics

**Screening and enrolment metrics for 41 sites**	
Months open per site	
Mean (SD)	27.3 (10.7)
Median (IQR)	28 (10)
Range	9–45
Cumulative screening months, *n*	1076
Screening months per site	
Mean (SD)	26.2 (10.3)
Median (IQR)	24 (11)
Range	9–45
Overall monthly recruitment rate	
Mean (SD)	52.5 (26.2)
Median (IQR)	57 (15)
Range	1–109
Main RCT patients recruited per site	
Mean (SD)	20.9 (13.2)
Median (IQR)	43 (36)
Range	3–170
Monthly recruitment per site	
Mean (SD)	2.04 (1.39)
Median (IQR)	1.74 (1.30)
Range	0.25–5.86
Number of patients recruited by ICU type	
Tertiary, *n* (%)	2203 (86.2)
Non-tertiary, *n* (%)	352 (13.8)

In this table, we present screening and enrolment metrics across a total of 45 months of the trial

*SD* standard deviation, *IQR* interquartile range

**Fig. 2:**
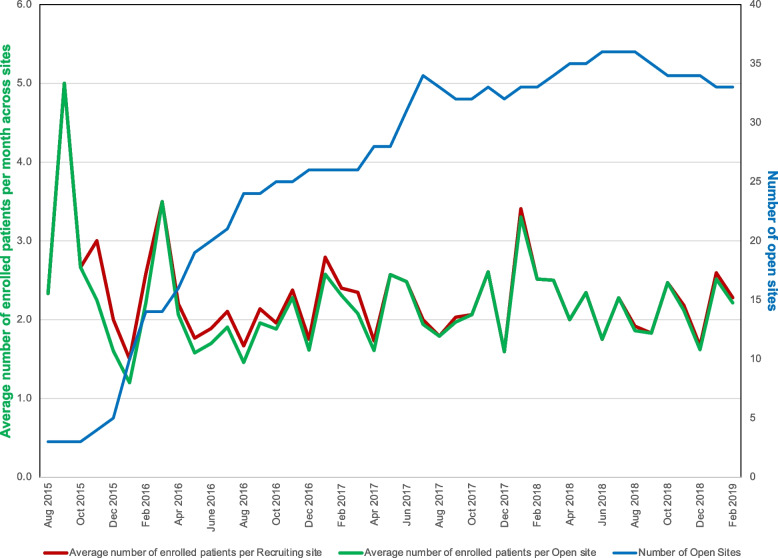
Average monthly enrolment across centres. This figure depicts the average number of patients enrolled per recruiting site (red) and per open site (green) with the total number of open sites (blue) over the course of the trial

In Fig. 2, we demonstrate average monthly enrolment across centers in ascending order, in addition to the number of recruiting sites and open sites. In the PROSPECT trial, 4.1% of total screening months were paused for various reasons including conference leave, holidays, local staff turnover, and sites focusing on other priorities (e.g., data entry to close out another trial). At each site, the mean (SD) total enrolment was 20.9 (13.2) patients per site with sites recruiting between 3 and 170 total patients during the trial.

**Fig. 3:**
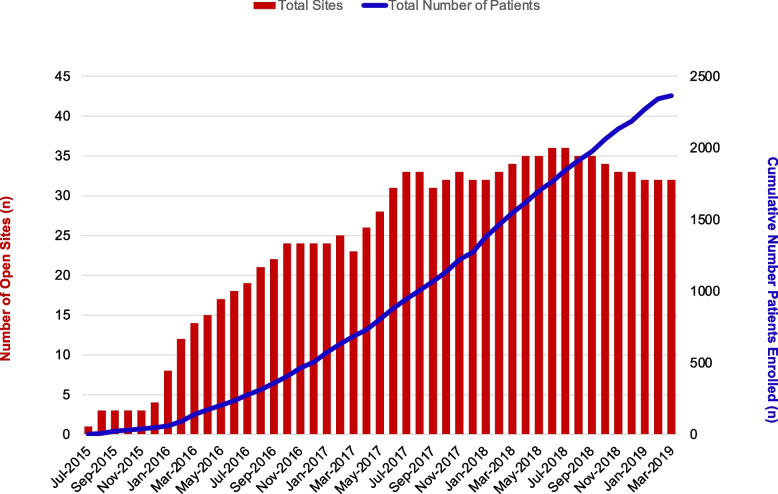
Cumulative patients enrolled. This figure depicts the cumulative number of patients enrolled by month in addition to the number of open sites over the course of the trial

In Fig. 3, we display cumulative patients enrolled in addition to number of open sites over the course of the trial. This reflects the intentional staggered startup of sites and steady enrolment increase.

### Aim 2: Factors associated with site enrolment rate

**Fig. 4:**
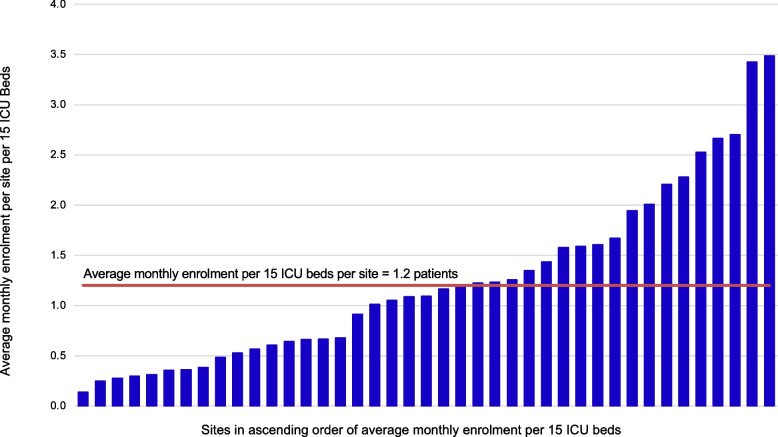
Average Monthly Enrolment Per Site Per 15 ICU Beds. This figure depicts the average monthly enrolment per site indexed to 15 ICU beds in ascending order. The red line indicates the overall average monthly enrolment rate per 15 ICU beds across sites (1.2 patients/month)

We summarize independent variables in Table 4. The mean (SD) time from regulatory approval to first enrolment was 69.1 (60.5) days. Sites engaged in the trial in stages based on methods center capacity to launch a center across the 45 open months; 16 (39.0%) engaged within the first quarter, 10 (24.4%) engaged in the second quarter, 13 (31.7%) engaged in the 3rd quarter, and 2 (4.9%) engaged in the final quarter of the enrolment period (Fig. 4). Of the 41 lead research coordinators, 18 (43.9%) had ≥ 10-year experience at the time of trial initiation.

**Table 4:** Description of variables evaluated for association with enrolment

**Variables**	
Lead research coordinator ≥ 10 years of experience, *n* (%)	18 (43.9)
Site engagement during 1st quartile, *n* (%)	16 (39.0)
Days from regulatory opening to first enrolment	
Mean (SD)	69.1 (60.5)
Median (IQR)	54 (29–87)

In this table, we describe characteristics related to research coordinator experience, site engagement in the trial during the first quartile of enrolment, and time from regulatory approval to first enrolment

*SD*, standard deviation; *IQR*, interquartile range

There was no association between time from regulatory approval to first enrolment or years of research coordinator experience with site-based enrolment rates in the negative binomial regression model (Table 5). However, enrolment rate was associated with the quartile of the trial period when a site engaged, such that sites participating in the first quarter of the trial recruitment was significantly associated with higher enrolment. Of the 16 sites which were engaged during the first quartile, 11 were original pilot trial sites.

**Table 5:** Factors evaluated for association with trial enrolment rate

Independent variables	Incidence rate ratio (95% CI)	*p*-value
Lead research coordinator ≥ 10 years of experience, yes vs no	0.72 (0.47, 1.10)	0.127
Site engagement during 1st quartile, yes vs no	1.62 (1.07, 2.47)	0.024
Days from regulatory approval to first randomized patient, based on a 30-day interval	0.93 (0.84, 1.02)	0.135

In this table, we present results of the negative binomial regression analysis evaluating the association between monthly enrolment rate and research coordinator experience, site engagement during the first quarter of trial enrolment, and time from regulatory approval to first enrolment

*CI* Confidence interval

## Discussion

In this analysis of a multicenter critical care trial, we observed heterogeneous duration and intensity of recruitment efforts across sites.

The variability in the number of active screening months across different sites reflects the diverse timelines in engaging patients for enrollment. Despite an overall rise in the number of open sites over time, the rate of patient enrollment exhibited no significant variation. This finding suggests that while early sites exhibit enthusiasm and capacity for patient recruitment, the involvement of later sites is crucial to maintaining a consistent enrolment rate. This underscores the importance of strategic planning and sustained effort throughout the entire duration of a multicenter trial, as sites launching a trial both early and late play key roles in achieving successful enrollment.

Sites joining in the first quartile of the trial had significantly higher monthly enrollment rates than those joining later. This may be explained through characteristics of early involved sites such as the participation of 11 of 16 sites in the first quarter which engaged in the pilot phase of this trial, site-driven enthusiasm for the research question, site investigators who were grant co-applicants, or had prior fruitful collaborations with the coordinating team [13]. Previous analyses have shown a positive association between a site having an investigator on the trial steering committee and that site’s recruitment [4]. Our findings build on prior analyses of a multicenter thromboprophylaxis trial wherein centers engaging in the first year had significantly higher consent rates, along with centers with more research staff and research coordinators having at least 10 years of experience [14].

Previous critical care trials have demonstrated asymmetric distribution of recruitment across sites, with a small number of ICUs recruiting a large proportion of patients [12]. In addition to results of the current analysis, these findings raise the question of whether enhanced mentorship of sites which engage later in a trial may improve overall trial enrolment efforts. Strategies to consider may include methodological and regulatory training for research coordinators beyond trial-specific site initiation visits, sharing best practices from sites engaged earlier, leveraging the experience of sites engaged earlier, mentorship on best practices from well-established sites, and regular communication opportunities that foster knowledge exchange. Implementing these proactive measures may assist with recruitment across all centers, regardless of site and stage of engagement in a trial.

We did not identify a significant association between monthly patient enrolment rate and time from study site start-up to first randomization. By contrast, the “time to enroll the first patient” has been a predictor of overall patient enrolment in multicenter randomized trials in other fields such as perinatal medicine, whereby factors most associated with above-average enrolment rates were related to a clearly defined “system” for recruitment, a dedicated research coordinator, engagement of other staff, and time from ethics approval to first patient enrolled [4, 15].

We did not document a relationship between years of research coordinator experience and enrolment rate, which has been previously shown to influence recruitment [14–16]. An association may not have been observed for this trial, related to the conventional threshold of one decade for assessing the relationship between research experience and enrolment rate rather than analyzing this variable in other ways such as median years of experience. These findings should be interpreted in light of frequent educational activities for both new and seasoned staff associated with the Canadian Critical Care Trials Group of Research Coordinators (e.g., educational webinars, annual training workshops, grants and travel awards for professional development).

Future investigations on determinants of enrolment should explore variables such as site-specific patient case-mix relative to inclusion and exclusion criteria, clinician engagement, local scientific priorities, workload disincentives, financial incentives, and local research infrastructure at participating sites.

## Strengths

In this study, the first aim to analyze patient enrolment per month across sites over the course of a trial was addressed by avoiding the confounding effect of number of ICU beds and duration of trial engagement, which would generate more enrolment opportunities, all else being equal. We avoided arbitrary cut-offs for high versus low recruiting sites. The second aim of examining factors during a site’s engagement which may be associated with monthly enrolment was analyzed within the context of a peer-review funded trial led by an established research consortium. Enrolment was complete in March 2019; thus, the results are not influenced by the coronavirus SARS-CoV-2 pandemic.

## Limitations

This retrospective study was limited by variables collected during the trial. Staggered site start-up was intentional by the methods center, and detailed site-specific determinants of the timing of engagement were not collected. Our sample size limits the number of factors admissible for adjusted analyses, and the possible influence of confounding factors cannot be excluded, such as daily research staff presence, the number, design and consent model of other trials operational during this period, and co-enrollment policies influencing a site’s research practices [4]. The enrolment patterns that are associated with enrolment rates may vary in other trials; for example, higher enrolment rates may be determined by other factors. The generalizability of the findings should be considered in light of 42 Canadian centers, 1 in the United States, and 1 in Saudi Arabia; results may vary in different settings. We cannot exclude the possibility of inequitable trial access creating selection bias in trial enrolment, as reflected in the inability to contact many substitute decision-makers, perhaps related to socioeconomic disparities in the catchment area of some hospitals, and other unmeasured factors.

## Conclusion

In summary, this analysis of enrolment rates within an ICU-based randomized clinical trial showed considerable variation in active screening months across sites. Adjusting for size of the ICU and duration of trial engagement, sites recruiting in the trial’s initial quartile had notably higher enrolment rates than other sites; however, research coordinator experience and time to first randomization were not significantly associated with enrolment rates. Our findings contribute to broader efforts to understand and optimize patient recruitment in critical care trials and highlight the complexity of recruitment dynamics and the need for tailored approaches to trial planning and execution.

## Data Availability

The datasets used and/or analyzed during the current study are available from the corresponding author on reasonable request.
